# Monkey hybrid stem cells develop cellular features of Huntington's disease

**DOI:** 10.1186/1471-2121-11-12

**Published:** 2010-02-05

**Authors:** Chuti Laowtammathron, Eric CH Cheng, Pei-Hsun Cheng, Brooke R Snyder, Shang-Hsun Yang, Zach Johnson, Chanchao Lorthongpanich, Hung-Chih Kuo, Rangsun Parnpai, Anthony WS Chan

**Affiliations:** 1 Yerkes National Primate Research Center, 954 Gatewood Rd, NE Atlanta, GA 39329, USA; 2 Department of Human Genetics, Emory University School of Medicine, 615 Michael St, Atlanta, GA 30322, USA; 3 Embryo Technology and Stem Cell Research Center, School of Biotechnology, Suranaree University of Technology, Nakhon Ratchasima 30000, Thailand; 4 Stem Cell Program, Institute of Cellular and Organismic Biology and Genomics Research Center, Academia Sinica, Taipei, Taiwan

## Abstract

**Background:**

Pluripotent stem cells that are capable of differentiating into different cell types and develop robust hallmark cellular features are useful tools for clarifying the impact of developmental events on neurodegenerative diseases such as Huntington's disease. Additionally, a Huntington's cell model that develops robust pathological features of Huntington's disease would be valuable for drug discovery research.

**Results:**

To test this hypothesis, a pluripotent Huntington's disease monkey hybrid cell line (TrES1) was established from a tetraploid Huntington's disease monkey blastocyst generated by the fusion of transgenic Huntington's monkey skin fibroblast and a wild-type non-transgenic monkey oocyte. The TrES1 developed key Huntington's disease cellular pathological features that paralleled neural development. It expressed mutant huntingtin and stem cell markers, was capable of differentiating to neural cells, and developed teratoma in severely compromised immune deficient (SCID) mice. Interestingly, the expression of mutant htt, the accumulation of oligomeric mutant htt and the formation of intranuclear inclusions paralleled neural development *in vitro *, and even mutant htt was ubiquitously expressed. This suggests the development of Huntington's disease cellular features is influenced by neural developmental events.

**Conclusions:**

Huntington's disease cellular features is influenced by neural developmental events. These results are the first to demonstrate that a pluripotent stem cell line is able to mimic Huntington's disease progression that parallels neural development, which could be a useful cell model for investigating the developmental impact on Huntington's disease pathogenesis.

## Background

Huntington's disease (HD) is an autosomal dominant neurological disorder caused when the CAG expansions encode the polyglutamine (polyQ) stretches at the N-terminus of the huntingtin (htt) protein [1]. HD is a devastating disorder that results in motor dysfunction, psychiatric disturbances and cognitive impairment. Typically, HD patients progress to their death 15 to 20 years after the onset of symptoms at mid-life. However, the age of onset is highly correlated to the size of polyQ, while CAG repeats below 37 are considered unaffected. Key neuropathological features can be found in the striatal region, specifically the medial spiny neurons where neurodegeneration can also be observed throughout the central nervous system. Unique HD pathology is characterized by the accumulation of oligomeric mutant htt, the formation of intranuclear inclusions (NIs), neuropil aggregates and progressive neuronal death.

Although htt plays a crucial role in early embryogenesis [2,3], the functions of htt remain largely unknown. The role of htt in neural development is intriguing since htt is widely expressed in the body with its highest levels of expression in the brain and testis, while the primary site of damages in HD are found in the brain [4-7]. In order to clarify the mechanism of neural specific degeneration and the impact of cell types on HD pathogenesis, pluripotent stem cells that are capable of differentiating into multiple cell lineages are a unique model for studying cell and tissue specific pathogenesis of HD.

Human HD-ES (hHD-ES) cell lines have been generated using human embryos [8] or by induced pluripotency using HD patients skin cells [9]. These hHD-ES cell lines are unique resources for studying HD; however, follow up study has been limited. Although hHD-ES cells carry mutant htt, the pathological sequence is expected to follow a similar time-course in HD patients, typically developing during mid-life. So far, no good HD cell model has yet been reported that develops hallmark HD cellular pathological features paralleling neural development. The latest development of transgenic HD monkeys suggests that N-terminal fragments of htt and expanded polyQ can accelerate the onset of HD in higher primates with distinctive neuropathological and cognitive behavioral characteristics [10]. The purpose of the study is to develop a pluripotent stem cell model for studying the mechanism of HD and the impact of developmental events on HD pathogenesis, which could also be used as a platform for drug discovery research and the development of new treatments. Because of the development of robust HD features in transgenic HD monkeys, we expect a pluripotent primate stem cell line with small *htt *fragments and expanded polyQ may lead to the development of hallmark HD cellular pathology that parallels neural development.

Although there is an immediate need for a pluripotent stem cell model that develops HD cellular phenotypes that parallel neural differentiation for studying HD pathogenesis, the use of nuclear transplantation derived stem cells has been limited by low efficiency [11]. While induced pluripotency is a promising method, only one study on induced pluripotent thesus macaque stem cells has been reported, which forced us to consider an alternative strategy to derive pluripotent HD monkey stem cells [12]. We have established a pluripotent HD monkey hybrid stem cell line, TrES1, that replicates the impact of mutant htt during the course of *in vitro *neuronal development. TrES1 was created by using a tetraploid embryo generated by the fusion of a transgenic HD monkey skin fibroblast with a wild-type non-transgenic (WT)-monkey oocyte. Using this TrES1, we have demonstrated the progressive development of HD hallmark cellular features that parallel neuronal development *in vitro *in higher primate pluripotent stem cells for the first time.

## Results

### Characterization of HD monkey skin fibroblast

**Figure 1:**
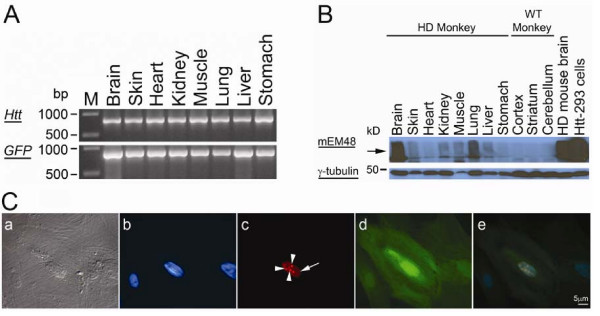
**Characterization of HD monkey and HD monkey skin fibroblasts**. (A) The presence of transgenes "mutant *htt *and *GFP*" in brain and peripheral tissues of HD monkey was confirmed by PCR analysis using primer sets specifically for mutant htt (top panel) and for the GFP gene (bottom panel). (B) Expression of the transgenic mutant HTT was confirmed by Western blot analysis in brain and peripheral tissues using mEM48 (top panel). Immunoblot revealed high-molecular-mass oligomeric HTT (arrow). The blot was also probed with an antibody to γ-tubulin as an internal control (bottom panel)., Wild-type (WT) non-transgenic monkey. (C) Immunostaining of primary cultured skin fibroblast of transgenic HD monkey using mEM48 demonstrated that transgenic mutant htt was distributed in the nuclei (arrow; C-c) and intranuclear inclusions (arrowheads; C-c) were also revealed. Expression of GFP was also revealed by epifluorescent microscopy (d). (C-a) transmission light image; (C-b) Hoechst DNA staining; (C-c) mEM48 staining; (C-d) epifluorescent light image of GFP; (C-e) overlay image. Scale bar = 5 μm.

Skin cells were isolated from a miscarried male transgenic HD monkey (rHD) at four months of gestation. rHD was confirmed transgenic with mutant *htt *and *GFP *gene by PCR (Figure 1A). A total of 72 CAG repeats were confirmed in the transgenic mutant *htt *gene, which was identical to the parent skin fibroblast.

The expression of mutant htt was confirmed by Western blot and immunohistochemistry with mEM48, a monoclonal antibody whose reaction with human *htt *is enhanced by polyQ expansion [10]. Western blotting of brain and peripheral tissues demonstrated the presence of oligomeric htt at high molecular weight (>250 kD) in the upper portion of a gradient polyacrylamide gel (Figure 1B; Arrow). Oligomeric mutant htt was presented in the peripheral tissues (Figure 1B) and brain (Figure 1B) of rHD but not in WT-monkeys. The extent of expression and aggregation levels of mutant htt was observed among peripheral tissues (Figure 1B), while only some skin cells developed htt aggregates and NIs (Figure 1C).

### Generation of HD monkey tetraploid embryo and derivation of a hybrid cell line

**Figure 2:**
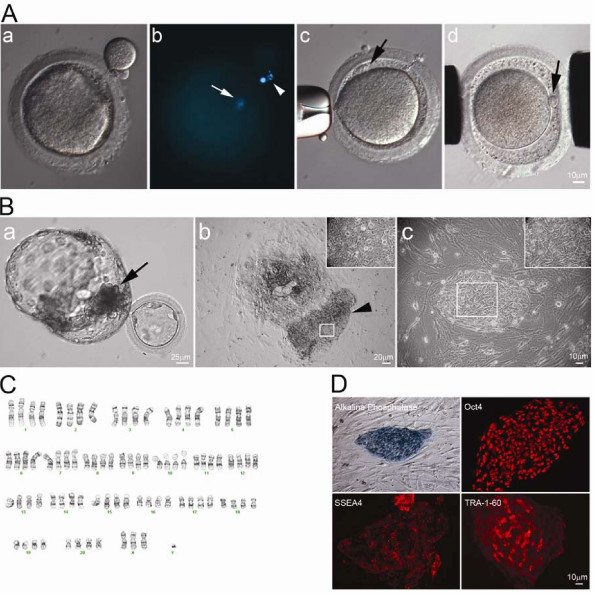
**Establishment of HD hybrid cell line**. (A) First polar body of mature rhesus macaque oocyte was removed by gentle squeezing through a slit of zona pellucida (A-a). Staining of 1^st ^polar body DNA (arrowhead) and oocyte DNA (arrow) (A-b). HD monkey skin cell was placed under the zona pellucida (black arrow) (A-c). Reconstructed oocyte with HD monkey skin cell (A-d; yellow arrow) was placed between two electrodes for electrofusion (A-d). (B) Day 12 hatching blastocyst derived from HD monkey hybrid embryo (B-a; arrow indicated ICM). HD monkey hybrid blastocyst outgrowth at six days after attached onto feeder cells (B-b). High magnification of selected region (inset) of the ICM outgrowth (arrowhead). HD monkey hybrid cell line (TrES1) at passage 10 (B-c). (C) G-banding analysis of TrES1. Cytogenetic analysis of TrES1 demonstrated tetraploid chromosome (84; XXXY). (D) Expression of ES-cell specific markers: Alkaline phosphatase, Oct4, SSEA4 and TRA-1-60.

The primary cultured skin cells of rHD were used to derive tetraploid embryos by fusion with mature WT-monkey oocytes. The first polar body (PB) was removed through a pre-cut zona-pellucida (ZP; Figure 2A-a and 2A-b) and a skin cell was placed under the ZP (Figure 2A-c) followed by electrofusion (Figure 2A-d) to create a hybrid embryo. The reconstructed hybrid embryos were chemically activated and cultured until blastocyst stage for the derivation of ES cells (Figure 2B).

Two out of four reconstructed HD monkey hybrid embryos were developed to blastocyst. The hybrid blastocysts (Figure 2B-a) were placed onto mouse fetal fibroblast (MFF) feeder cells and allowed to form an outgrowth (Figure 2B-b). At 14 to 16 days, one of the blastocysts developed an outgrowth with ES cell like morphology (large nucleus and a high nuclear to cytoplasmic ratio) (Figure 2B-b). An ES cell like region was mechanically dissected and cultured. The resultant HD monkey hybrid cell line, named TrES1, retains monkey ES cell morphology (Figure 2B-c) and is pluripotent. Cytogenetic analysis confirmed that TrES1 is a tetraploid hybrid cell line with three "X" chromosomes and one "Y" chromosome (Figure 2C), which suggested that a set of "XY" chromosome was derived from skin cell of rHD while a set of "XX" chromosome was derived from the monkey oocyte.

Inheritance of mutant *htt *and *GFP *genes in TrES1 was confirmed by PCR analysis while these transgenes can only be derived from rHD but not from the WT-monkey oocyte.

### Genetic identity analysis

Microsatellite analysis and comparison of its mitochondrial sequence were used to determine the genetic identity of TrES1. In all genotyping assays, all alleles presented in HD monkey skin cells and the lymphocytes of oocyte donor were also presented in TrES1 (Table 1). This suggested that TrES1 is a tetraploid and contain the nuclear genetic material of both the rHD and oocyte donor, thus TrES1 is a true hybrid cell line.

**Table 1:** Microsatellite analysis of monkey hybrid stem cells

**Locus**	**D9s261**	**D19s582**	**D16s403**	**D4s413**	**D5s108**
**Donor**	96/105	158/170	171/177	141/152	191/193
**Recipient**	103/105	167/175	167/169	141/150	179/189
**Hybrid**	96/103/105	158/167/170/175	167/169/171/177	141/150/152	179/189/191/193
**Locus**	**D2s146**	**D3s1768**	**D6s493**	**D7s513**	**D13s1371**
**Donor**	213/221	230/230	272/328	195/208	145/153
**Recipient**	208/210	210/226	266/269	193/193	169/174

Genotypes for 10 loci were assayed on genomic DNA of HD monkey skin cells, lymphocyte of monkey oocyte donor and TrES1. All alleles present in the skin fibroblasts and oocyte donor are presented in TrES1, indicating TrES1 is a hybrid of HD monkey skin cell and monkey oocyte.

For DNA comparisons of the mitochondrial sequence, 16 rhesus macaque specific single nucleotide polymorphisms (SNPs) were analyzed. In all 16 cases the TrES1 matched the oocyte donor but not the rHD skin cells (Table 2), conclusively showing that the mitochondria present within the hybrid line were inherited from the female monkey who donated the oocyte that created the hybrid embryo that was used for the derivation of TrES1. This result is consistent with a prior study in somatic cell nuclear transplantation (SCNT) that mitochondria inheritance of reconstructed embryos is primarily derived from recipient oocytes instead of the donor cell nuclei [11].

**Table 2:** Mitochondrial sequence analysis of monkey hybrid stem cells

	*	*	*	*
Donor	TTG **G **CA	CAA **A **CA	CTA **C **AA	CAA **G **AGG
Recipient	TTG **A **CA	CAA **G **CA	CTA **T **AA	CAA **C **AGG
Hybrid	TTG **A **CA	CAA **G **CA	CTA **T **AA	CAA **C **AGG

Mitochondrial sequence comparisons of HD monkey skin cells, lymphocyte of monkey oocyte donor and TrES1. The representative example is position 371-731 of Macaca mulatta NCBI reference sequence NC_005943. Highlighted regions clearly show mitochondrial inheritance of TrES1, is obtained from the monkey oocyte.

### Stem cell properties and pluripotency

To determine the stem cell properties of TrES1, immunostaining of common monkey ES cell markers was used. TrES1 expressed alkaline phosphatase (AP), Oct4, and stem cell specific surface antigens (SSEA4 and TRA-1-60; Figure 2D).

**Figure 3:**
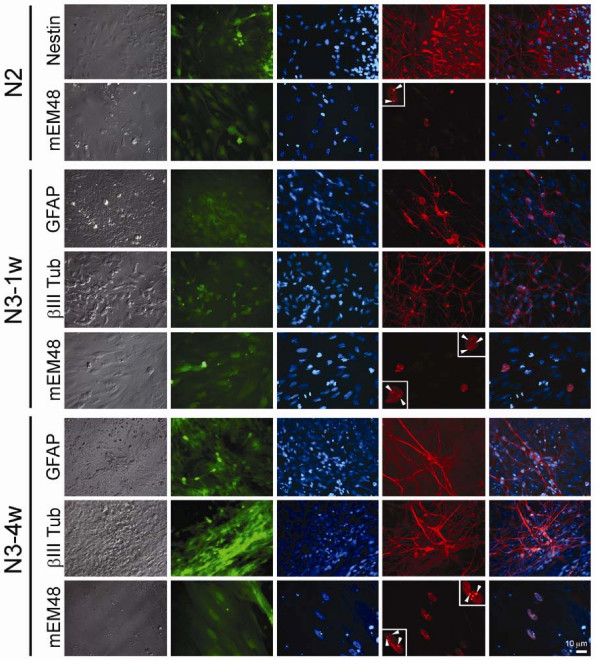
**Immunocytochemical analysis of *in vitro *differentiated TrES1**. TrES1 was differentiated toward neuronal lineage *in vitro *using a step-wise differentiation protocol. Antibodies specific for neural progenitor cells (nestin), glial fibrillary acidic protein (GFAP), and mutant htt (mEM48) were used for immunostaining at different differentiation stages: N2, N3-1 week (N3-1w), and N3-4 weeks (N3-4w). At N2 stage, all cells were stained with Nestin and some were stained positive with mEM48. At N3-1w and N3-4w, cells were stained with GFAP, βIII-tubulin and mEM48. First column-brightfield images; second column-epifluorescent images of GFP; third column-DNA staining with Hoechst; fourth column-immunostaining with specific antibodies, and fifth column-overlay images of the third and fourth columns. Insets are images of selected nuclei with nuclear inclusions at higher magnification.

To determine the pluripotency of TrES1, *in vitro *differentiation to neural cells was performed. A step-wise differentiation protocol was used in this study while immunostaining was performed at different stages to confirm successful differentiation [13]. The expression of nestin was observed at N2 stage when selective expansion of neural progenitor cells (NPCs) occurred (Figure 3). In general, one week induction for neuronal maturation was suggested at N3 stage (N3-1w). In order to mimic mature neurons in adult brains that are primarily maintained at a post-mitotic stage, we have extended N3 culture to four weeks (N3-4w) to determine if extended culture impacted the mutant htt associated phenotype. Glial fibrillary acidic protein (GFAP), a glial cell marker and neural specific βIII tubulin, was detected by immunostaining at both N3-1w and N3-4w stages (Figure 3), which suggested TrES1 was capable of differentiating to mature neuronal cell types. Although the expression of mutant htt does not seem to affect neural differentiation of TrES1, potential effects of mutant htt on differentiation toward specific neuronal or peripheral cell types cannot be excluded and further investigation is necessary. Furthermore, the expression of GFP was observed at all stages (Figure 3).

### Expression of mutant htt in TrES1 derived neuronal differentiation

To determine if the expression of mutant htt and the development of HD specific cellular pathology are related to the course of neural development, the expression patterns of mutant htt, the accumulation of mutant htt aggregate, the presence of oligomeric mutant htt and the formation of NIs were examined by quantitative real time PCR (Q-PCR), Western blot, immunostaining and cell count at various stages during *in vitro *development.

**Figure 4:**
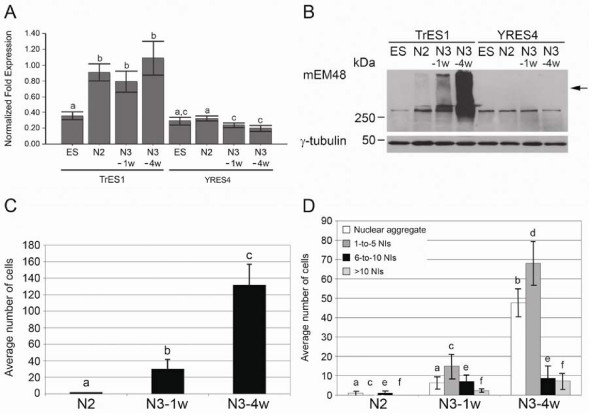
**Expression pattern of mutant htt in neural differentiated TrES1**. (A) Expression levels of mutant htt at various developmental stages were determined by Q-PCR. YRES4 is a WT-monkey ES cell line and was used as a control. The expression levels of mutant htt in differentiated TrES1 were significantly increased at N2, N3-1w and N3-4w compared to undifferentiated TrES1 (ES) and YRES4 at all differentiation stages. Columns with the same letter indicate no significant difference (P > 0.05). (B) Western blot analysis using mEM48 revealed a gradual increase of oligomeric transgenic mutant htt as TrES1 progresses during neural differentiation (N3-4w > N3-1w > N2) whereas no high molecular weight mutant htt aggregates was detected in undifferentiated TrES1 or YRES4 at all stages. (C) Increase of cells expressing mutant htt detected by mEM48 was observed as differentiation progresses. Columns with the same letter indicate no significant difference (P > 0.05). (D) Expression pattern of mutant htt was categorized into four groups: soluble form, 1-to-5 nuclear inclusions (NIs), six to10 NIs, and more than 10 NIs. Columns of the same category with the same letter indicate no significant different (P > 0.05).

Q-PCR analysis revealed similar expression levels of mutant htt in undifferentiated TrES1 and YRES4 (WT-monkey ES cells) at different differentiation stages (Figure 4A). A significant increase in the expression of mutant htt was observed in TrES1 at N2, N3-1w and N3-4w when compared to undifferentiated TrES1 and YRES4 at respected stages (Figure 4A). However, no difference was observed in TrES1 at N2, N3-1w and N3-4w (Figure 4A). The same batch of cell samples was then used for Western blot analysis. Oligomeric mutant htt was revealed in TrES1 at N2, N3-1w and N3-4w stages but not in undifferentiated TrES1 and YRES4 at any stages (Figure 4B). Furthermore, the extent of oligomeric mutant htt was gradually enhanced as TrES1 progressed during *in vitro *neuronal differentiation (Figure 4B). While the accumulation of oligomeric mutant htt increased in differentiating TrES1, oligomeric mutant htt was substantially increased in N3-4w compared to N3-1w (Figure 4B). This result suggests the possible impact of neural development on HD pathogenesis.

In order to determine the impact of mutant htt and the extent of cellular pathology during the course of neural development, undifferentiated TrES1, TrES1 at N2, N3-1w and N3-4w were immunostained with mEM48. Cells developing mutant htt aggregates and containing NIs were identified and counted. While the expression of mutant htt was not detected in undifferentiated TrES1 by immunostaining, the number of mEM48+ cells was significantly higher in N3-4w (32.2%; 132 ± 42.5; n = 1484) > N3-1wk (8.4%; 30.3 ± 19.4; n = 1078) > N2 (0.26%; 2 ± 0; n = 1271) (Figure 4C). The mEM48+ cells were then grouped as cells that form nuclear aggregate, cells with nuclear aggregate and contained one-to-five, six-to-10, and more than 10 NIs (Figure 4D). The number of TrES1 with nuclear aggregate and developing one to five pieces of NIs was significantly increased in N3-4w compared to N3-1w and N2. This finding was consistent with the Q-PCR and Western blot analysis, which suggested the expression of mutant htt was not different between N2, N3-1w and N3-4w, but the accumulation of oligomeric mutant htt increased as TrES1 continued neuronal differentiation *in vitro *and extended culture.

### In vivo differentiation of TrES1 in the striatum of SCID mice

**Figure 5:**
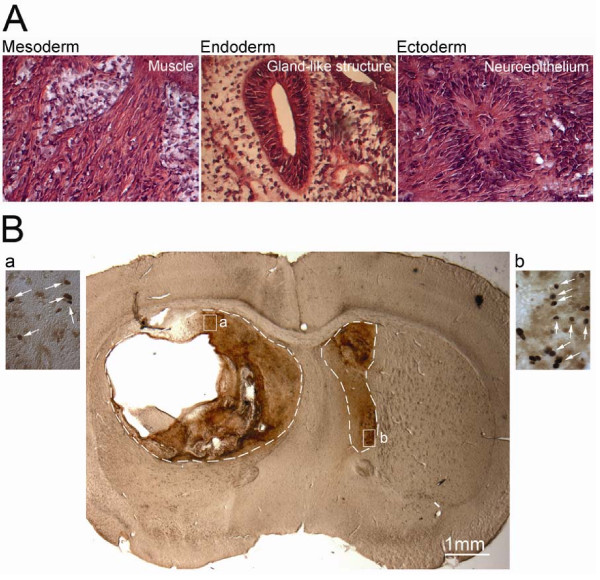
**Teratoma derived from TrES1 and expression of mutant htt in striatal graft of TrES1**. Undifferentiated TrES1 and TrES1 derived NPCs were implanted into the striatum of SCID mice and recovered at six weeks for morphological and immunohistochemical analysis. (A) Hematoxylin and eosin staining of teratoma derived from undifferentiated TrES1. (B) Undifferentiated TrES1 (Left hemisphere) and TrES1 derived NPCs (Right hemisphere) were implanted into contralateral hemispheres of SCID mice. Immunohistochemical staining using mEM48 revealed the expression of mutant htt in both hemispheres (B-a and B-b). Areas surrounded by interrupted line indicated the locations of the cell graft.

**Figure 6:**
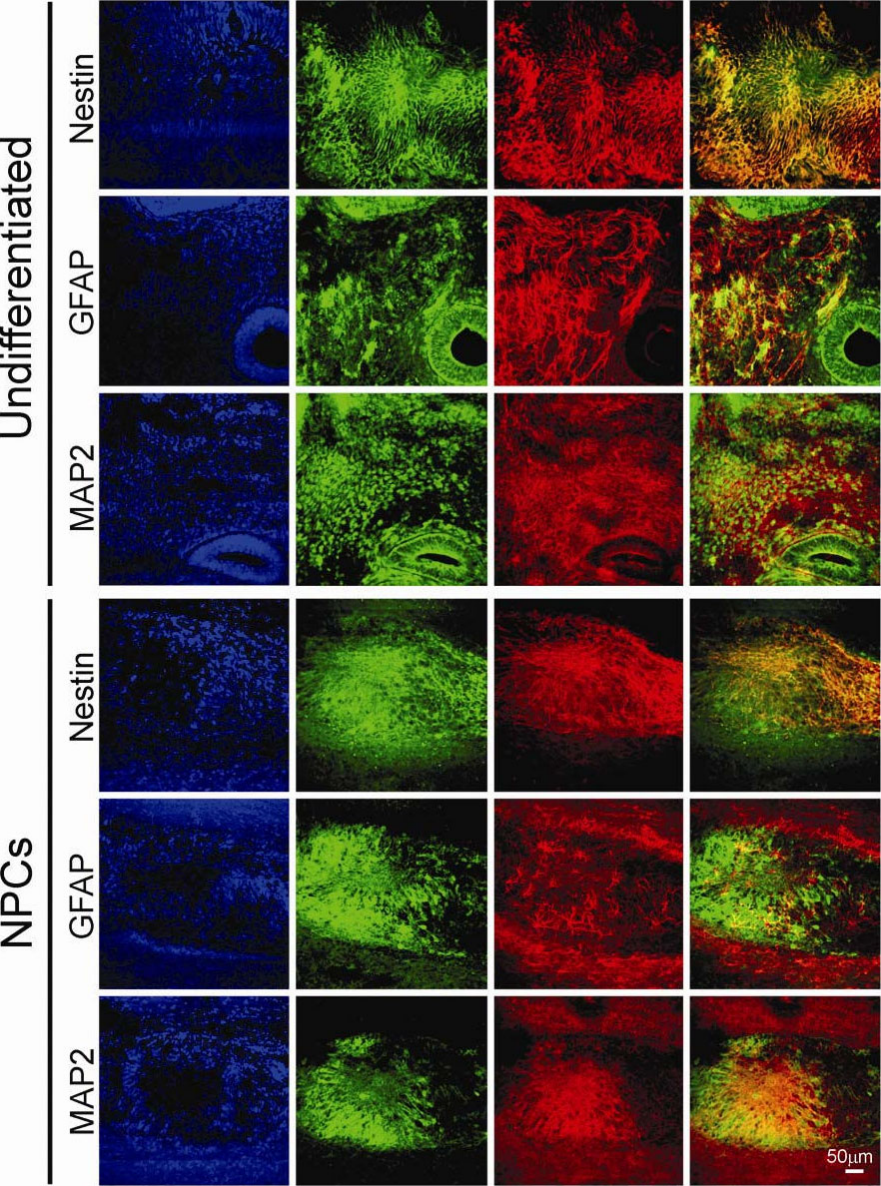
***In vivo *differentiation of TrES1**. Undifferentiated TrES1 and TrES1 derived NPCs were implanted into contralateral hemispheres of SCID mice for six weeks. Nestin, GFAP and MAP2 were co-expressed with GFP in both hemispheres with the TrES1 graft while homogenous expression pattern was observed at the NPCs implanted hemisphere. First column-DNA staining; Second column-epifluorescent images of GFP; Third column-Immunostaining using specific antibodies; Fourth column-overlay images of second and third column. Scale bar = 50 μm.

To determine the pluripotency of TrES1 *in vivo*, undifferentiated TrES1 and TrES1 at the N2 stage (presumably NPCs) were implanted into the striatum of the contralateral hemisphere of severely compromised immune deficient (SCID) mice. At four-to-10 weeks post-implantation, animals were euthanized and their brains were recovered for morphological analysis (Figure 5) and an immunohistochemistry study using different antibodies to determine neural differentiation (Figure 6) and the expression of mutant htt (Figure 5B).

A histological study showed that only the undifferentiated TrES1 implanted hemisphere developed teratoma, which contained different tissue types, including gut-like epithelium (endoderm), muscle (mesoderm) and neural tissues (ectoderm) (Figure 5A). No teratoma was observed at the contralateral hemisphere where NPCs were implanted (Figure 5B). On the other hand, an immunohistochemical study revealed the expression of neuronal markers including nestin, GFAP and microtubule-associated protein (MAP2) (Figure 6) in both hemispheres. Consistent with histological analysis, the hemisphere that was implanted with undifferentiated TrES1 developed teratoma with heterogenous expression of neuronal markers suggesting non-neuronal tissues was developed (Figure 6). In contrast, the hemisphere implanted with TrES1 derived NPCs homogenously expressed neuronal markers and was co-labeled with GFP that was only expressed in TrES1 and the derivative cells (Figure 6). Similar results were observed in all SCID mice implanted with both cell types. Although NIs were not observed, a nuclear aggregate was observed in both hemispheres (Figure 5B-a and 5B-b).

## Discussion

In this study, we showed that TrES1, a hybrid cell line of Huntington monkey skin fibroblast and monkey oocyte, is pluripotent and develops robust HD cellular features as it progresses during neural development *in vitro*. The accumulation of mutant htt aggregates and the formation of NIs were significantly enhanced and increased at later stages of neural development while a relatively lower expression of mutant htt was detected in undifferentiated TrES1 with no detectable accumulation of mutant htt aggregates and NIs. Our finding is consistent with HD pathogenesis where neuronal tissues are the primary targets and post-mitotic neural cells accumulate oligomeric mutant htt as disease progresses, whereas peripheral cell and tissue types are expected to have minimal impact.

A pluripotent cell line with an inherited genetic disorder is one of the best models for understanding the underlying mechanism of developmental events in disease progression [8,9,14,15]. Multipotent differentiation capabilities of pluripotent cells are particularly intriguing for the study of neurodegenerative diseases such as HD, because pathological events specifically target neuronal cell types where peripheral tissues rarely develop comparable cellular pathology [6,10,16,17] TrES1 is capable of differentiating into neuronal cell types that mimic early HD developmental events. Unlike other HD cell models, either by transient or stable expression of mutant htt in somatic cells (CHO and 293) or a neuronal cell line (PC12) [18-21], a pluripotent cell line is capable of replicating the influence of developmental events and mutant htt on HD pathogenesis that no other cell model can achieve. Although HD mouse ES cells [15,22-25] and hHD-ES cells have been established [8,9], most of these cell lines do not develop robust HD cellular features that parallel neural differentiation, and detailed characterization of HD pathogenic features in hHD-ES cells has not been reported. Therefore, a pluripotent cell line such as TrES1, which develops key HD cellular phenotypes, is a unique cell model for studying HD and understanding fundamental differences between neuronal and peripheral cells/tissues in HD pathogenesis. Thus unique cell/tissue specific components and events that lead to differential susceptibility of HD cellular pathogenesis can be identified. One of the major concerns of deriving pluripotent stem cells such as TrES1 by tetrapolid technique is its potential instability due to the nature of tetraploidy. Thus the development of diploid HD stem cell lines from diploid embryos or by mean of nuclear transplantation and iPS technology is important for future applications such as cell therapy.

The impact of developmental events on the progression of HD was further suggested by the gradual increase of the aggregate form of mutant htt as neural differentiation progresses while the expression levels of mutant htt remains. The continued accumulation of mutant htt aggregate and the increase of cells with intranuclear inclusions in extended neuronal culture further suggest the potential impact on post-mitotic neural cells. While this study is the first step in characterizing HD monkey pluripotent stem cells, future development of a differentiation protocol toward specific neuronal cell types and peripheral cell types will facilitate the investigation of mutant htt cell type specific pathogenesis.

Due to ethical reasons, the development of pluripotent human hybrid cell lines is not an option. Recent success in developing iPS cells using skin cells of human patients [9,14]and monkeys [12] has opened a new door for investigating the potential of personal stem cells. The present study evolved from our latest success in developing a transgenic HD monkey model. While efforts in developing alternative methods for deriving pluripotent stem cell lines from HD monkeys continue, TrES1 provides a unique model for investigating the mechanism of HD pathogenesis and the role of neural developmental events. Furthermore, a pluripotent cell line such as TrES1, which develop hallmark HD features paralleling neural development, is a useful tool for accurate interpretation of therapeutic efficacy of new molecules and compounds. So far, there is no other cell model that replicates key HD cell pathology in parallel with the progression of neural development *in vitro*.

One possible explanation for the robust HD cellular phenotypes in TrES1 could be due to the over-expression of small htt fragments with expanded polyQ. HD monkeys that carried similar htt mutants developed HD clinical features early in life [10], which is consistent with our findings in TrES1. Thus stem cell lines derived from hHD patients by either traditional methods using PGD diagnosed embryos or iPS may not develop robust HD phenotypes comparable to TrES1 even with expanded polyQ because of the full-length htt. Studies in HD mouse models further support our speculation that full length htt is less toxic compared to small htt fragments [26-28]. Thus HD patients' derived stem cell lines may not be able to develop hallmark cellular features without extended culture time to allow the accumulation of cellular defects.

While a hybrid cell line is not a perfect model, we have now demonstrated that a pluripotent primate stem cells could replicate some of the key pathological features of HD suggesting the continue effort in developing a personal stem cell from HD patients by mean of induced pluripotency or other methods is of great value as a model for studying HD or as a cell source for therapy. However, the progression of HD phenotypes in such cell lines may vary because of the constitution of the mutant *htt *gene and human cell lines with full length htt and extended CAG repeat may require additional time to develop pathological features of HD.

## Conclusions

A pluripotent tetraploid Huntington's monkey stem cell line (TrES1) was derived by the fusion of transgenic HD monkey skin cell and monkey oocyte. TrES1 is the first primate stem cells that develop key HD cellular features (accumulation of mutant htt aggregate and the formation of intranuclear inclusions) paralleling *in vitro *neural development. Because of the robust development of HD phenotypes, TrES1 could be a useful tool for studying the developmental impact HD and as a platform for drug discovery research.

## Methods

### Regimen of follicular stimulation

Female rhesus monkeys exhibiting regular menstrual cycles were induced with exogenous gonadotropins [29,30]. The expression of monkey endogenous gonadotropins was down regulated at the beginning of mensis (day one to day two) by daily subcutaneous injections of Gonadotropin-releasing hormone (GnRH) antagonist (Antide; Ares Serono, 0.5 mg/kg body weight) for six days and by twice daily injection of recombinant human follicle-stimulating hormone [r-FSH: Organon Inc. 30 IU, intramuscular injection (i.m.)] concurrently. This was followed by the injection of r-FSH + recombinant human luteinizing hormone (r-hLH; Ares Serono; 30 IU each, i.m., twice daily) on the last three days. Ultrasonography was performed on day seven of the stimulation to confirm follicular responses. An i.m. injection of 1,000 IU recombinant human chorionic gonadotropin (r-hCG; Ares Serono,) was administered for ovulation induction when there were follicles at 3-4 mm in diameter. In general, r-hCG was administered at approximately 37 hours prior to oocyte retrieval for optimal maturation of metaphase II arrested oocytes.

### *In vitro *Maturation (IVM)

Oocytes were matured in modified CMRL-1066 containing 10% heat-inactivated fetal bovine serum (FBS; Hyclone Laboratories Inc., Logan, UT) supplemented with 40 μg/mL Sodium pyruvate, 150 μg/mL Glutamine, 550 μg/mL Calcium lactate, 100 ng/ml estradiol and 3 ug/ml of Progesterone for up to 36 hours in 35-μl drops of medium under mineral oil at 37°C with 5% CO_2_, 5% O_2 _and 90% N_2_.

### Generation of transgenic HD monkeys

High titer lentiviruses carryiing (1) exon 1 of human *htt *gene with 84 CAG repeats and (2) green fluorescent protein (*GFP*) gene under the regulation of human polyubiquitin C promoter, were injected into the PVS of metaphase II (MII) arrested monkey oocytes followed by intracytoplasmic sperm injection (ICSI) [10]. The resultant embryos were transferred into surrogate females for the generation of transgenic monkeys. Transgenic status was confirmed by PCR.

### Characterization and preparation of donor skin cells

Donor skin cells were primary cultures of skin tissue derived from miscarried transgenic HD monkey (rHD) at four months of gestation. The transgenic status of the skin cells was confirmed by PCR, immunostaining and Western analysis [10].

### Production of transgenic HD monkey tetraploid embryos

MII arrested oocytes were placed in TL-HEPES [31] with 5 μg/ml of cytochalasin B (Sigma) for 15 minutes. The 1^st ^polar body (PB) was gently squeezed out through a small slit at the zona pellucida (ZP). After thorough washes of the oocytes, skin cell was placed under the ZP. The couplet was fused by electrofusion using fusion electrodes in 0.3 M Manitol fusion medium (two direct currents, 30 volts 30 μsec; Electro cell fusion system LF-101, Nepa Gene Company). The reconstructed embryos were cultured in medium supplemented with 50 nM trichostatin A (TSA; Sigma) for 10-12 hours. Two hours after fusion, the reconstructed embryos were activated by 5 μM Ionomycin for five minutes and then incubated in 2 mM 6-Dimethylaminopurine (6-DMAP; Sigma) for five hours at 37°C with 5% CO_2_, 5% O_2_, 90% N_2_. The reconstructed embryos were further cultured in HECM 9 medium for eight days with 5% FBS added on Day two of culture. Fresh medium was replaced every two days.

### Establishment and maintenance of Huntington's monkey ES cells from tetraploid blastocyst

Tetraploid blastocysts were cultured for ten to 14 days until attached onto MFFs to form an outgrowth. The outgrowths, the exhibited prominent stem cell morphology, were mechanically removed, transferred onto freshly prepared MFFs and continued to culture for the derivation of monkey ES cells. Monkey ES cells were cultured in medium composed of knockout-Dulbecco's modified Eagle's medium (KO-DMEM) supplemented with 20% Knock-out Serum Replacement (KSR; Invitrogen), 1 mM glutamine, 1% non-essential amino acids and supplemented with 4 ng/ml of human basic fibroblast growth factor (bFGF; Chemicon). The HD monkey ES cells derived from tetraploid HD monkey embryos were named, TrES1.

### Transgenic status of the HD monkey ES cells

For detecting the *htt*-84Q gene, ubiquitin forward primer (5'-GAGGCGTCAGTTTCTTTGGTC-3') and *htt*-84Q-R reverse primer (5'-GCTGGGTCACTCTGTCTCTG-3') were used to yield an 818-bp product after amplification of genomic DNA from the HD monkey tissues. Genomic DNA (100 ng) from different tissues were subjected to PCR for 35 cycles at 96°C for 5 min, 96°C for 45 sec, 62°C for 45 sec, and 72°C for 150 sec, followed by 72°C for 7 min. To determinate the number of CAG repeats in HD monkeys, the PCR products were sequenced using HD exon 1-F primer (5'-GGCGACCCTGGAAAAGCTGA-3'). For GFP gene, ubiquitin forward primer (5'-GAGGCGTCAGTTTCTTTGGTC-3') and GFP-R reverse primer (5'-TAGTGGTTGTCGGGCAGCAG-3') were used for amplification for 35 cycles at 94°C for 5 min, at 94°C for 30 s, 64°C for 30 s, and 72°C for 20 s, followed by 72°C for 5 min, which yielded a product of 869 bp. DNA from WT-monkeys was used as the negative control, and plasmid *htt*-84Q and *GFP *were used as the positive controls.

### Genotyping

Genotyping was executed using a panel of 13 microsatellites, known to be highly polymorphic and possessing high levels of heterozygosity in other rhesus macaque populations [32,33]. Primers for each microsatellite were obtained with one of the standard Applied Biosytems (AB) five-dye labels. Amplification reactions were performed on AB 9700 thermal cyclers using MgCl_2 _concentrations of either 1.5 mM or 2.0 mM. Electrophoresis was carried out using an AB 3730 genetic analyzer, with all subsequent genotyping analysis using Genemapper 4.0. All genotyping was performed blind, with a positive and negative control included for each reaction.

### Immunostaining of mutant htt

For cell samples, differentiated TrES1 were fixed using 4% paraformaldehyde (PFA) for 15 mins. Then they were permeabilized and blocked. The sample was next incubated with primary antibody mEM48 (1:50) followed by incubation with secondary antibody conjugated with Alexa Red (Molecular Probe). DNA was counterstained with Hoechst 33342 (5 μg/ml), mounted in Vectashield antifade solution (Vector Labs), and sealed with nail polish. The specimen was examined with an epifluorescent microscope. For mouse brains, the mice were anesthetized and perfused using 4% PFA. Brain tissues were post-fixed in 4% PFA overnight at 4°C, transferred to 30% sucrose, stored at 4°C, embedded in Optimal Cutting Temperature (OCT) medium (Sakura) and cut at 50 μm, followed by DAB staining. For DAB staining, sections were incubated with 0.3% H_2_O_2 _for 15 mins, blocked for one hour, and incubated with mEM48 (1:50) at 4°C overnight. After washing with DPBS, the brain sections were processed with avidin-biotin using the Vectastain Elite ABC kit (Vector Laboratories), and immediately stained with DAB (Vector Laboratories) for 30-40 secs. Brain sections were mounted on the slides with mounting media (Sigma), and images were examined and captured by MetaMorph software (Universal Imaging).

### Immunostaining of stem cell markers

TrES1 were placed onto MFF in a four-well plate followed by two to three days culture, and was then fixed in 4% PFA, permeabilized by 1% Triton-X (excluded for cell surface markers), blocked with 2% BSA and 130 mM glycine in phosphate buffer saline (PBS). After overnight incubation with primary antibodies [Oct4 (Santa Cruz Biotechnology), SSEA-4 (Chemicon), TRA-1-60 (Chemicon)] followed by thorough washes, a secondary antibody conjugated with Alexa Red (Molecular Probe) was used for detection of the primary antibodies. DNA was counterstained with Hoechst 33342 (5 μg/ml). The specimen was examined with an epifluorescent microscope. Alkaline phosphatase assay was performed following manufacturer's instruction (Vector Lab).

### Quantitative RT-PCR (Q-PCR) of stemness factors

The total RNA of cell samples was extracted using RNeasy Mini Kit (Qiagen). RNA quality was determined by BioPhotometer (Eppendorf). Reverse transcription was performed by using High Capacity cDNA Reverse Transcription Kit (Applied Biosystems), and the resulted cDNA was used for Q-PCR. 2× Power SYBR^® ^Green PCR Master Mix (Applied Biosystems) was mixed with specific primers and cDNA, and subjected to the iQ5 real-time PCR detection system (Bio-Rad). for one cycle at 96°C for 12 mins; then at 96°C for 15 secs and 60°C for 30 secs for 50 cycles. The specific primers for mutant htt specific primers were: HD Exon 1-F: ATGGCGACCCTGGAAAAGCT and HD Exon 1-R: TGCTGCTGGAAGGACTTGAG. The specific primer for 18S: 18S F: CGGCTACCACATCCAAGGAA and 18S R: CCTGTATTGTTATTTTTCGTCACTACCT. Specific-qPCR primer sets targeting stem cell markers were: Oct 4 (Oct4-F: 5'-GCA ACC TGG AGA ATT TGT TCC T-3' and Oct4-R: 5'-GGG CGA TGT GGC TGA TCT-3'), Sox2 (Sox2-F: 5' GCA GGT TGA CAT CGT TGG TAA T-3' and Sox2-R: 5'CCC CCC GAA GTT TGC TGC G 3'), Nanog (Nanog-F: 5'-TGA AGC ATC CGA CTG TAA AGA ATC-3' and Nanog-R: 5'-CAT CTC AGC AGA AGA CAT TTG CA-3').

### Mitochondria Inheritance Analysis

Sequencing primers were designed in primer 3 http://frodo.wi.mit.edu/ in order to amplify two regions of rhesus mitochondrial DNA (*Macaca mulatta *NCBI reference sequence NC_005943). PCRs were performed using standard amplification reactions on AB 9700 thermal cyclers using 2.0 mM MgCl_2 _concentration. PCR products were checked for expected size by electrophoresis on agarose gels. Shrimp alkaline phosphatase and Exonuclease I were added to remove single strand DNA. Sequencing reactions were done using AB Big Dye terminator on a 9700 thermal cycler. The reaction was purified and sequencing reactions were performed on an AB 3730 genetic analyzer. Subsequent analysis was done using SeqScape genetic software. Positive and negative controls were sequenced along with experimental samples for each region.

### Cytogenetic analysis/G-banding analysis

TrES1 at passage 25 was treated with KaryoMax^® ^colcemid (Invitrogen) for 20 mins, dislodged with 0.05% Trypsin-EDTA, centrifuged and resuspended in hypotonic 0.075 M KCl solution for 20 mins. Following centrifugation, the cells were fixed three times in a 3:1 ratio of methanol to glacial acetic acid. The cell pellet was resuspended in 1 ml of fixative and stored at 4°C. For GTL-Banding, the fixed cell suspension was dropped on wet slides, air dried, and baked at 90°C for one hour. Slides were immersed in 0.5× Trypsin-EDTA (Invitrogen) with two drops of 67 mM Na_2_HPO_4 _for 20 to 30 secs, rinsed in distilled water and stained with Leishman Stain (Sigma) for 90 secs. Twenty metaphases were analyzed for numerical and structural chromosome abnormalities using an Olympus BX-40 microscope. Images were captured, and at least two cells were karyotyped using the CytoVysion^® ^digital imaging system (Applied Imaging).

### In vitro differentiation to neuronal lineage

TrES1 cell clumps were cultured in suspension for seven days for the formation of embryoid bodies (EBs). EBs were then allowed to attach onto a gelatin coated plate and cultured in N1 medium for seven days, N2 medium for 14 days and N3 medium for seven days to allow for differentiation into mature neuronal cell types. A four weeks extended culture at N3 stage was added to enhance maturation of neurons and mimic post-mitotic condition in the brain. The N1 medium was composed of KO-DMEM (Invitrogen) supplemented with minimum essential amino acid (Invitrogen), 200 mM of L-glutamine (Invitrogen) and N2 supplement (Invitrogen). The N2 medium was composed of N1 medium supplemented with 20 ng/ml bFGF. The N3 medium was composed of KO-DMEM supplemented with 1% FBS (Hyclone) and B27 supplement (Invitrogen). NPCs were immunostained with nestin, whereas successful differentiation of neuronal cell types was confirmed by the expression of neuron specific βIII tubulin and MAP2 [13].

### Counting of TrES1 with nuclear aggregate and NIs

After immunostaining with mEM48, each sample was examined and images were taken at different regions of the culture. A total of three 35 mm dishes of differentiated TrES1 at different differentiation stages (N2, N3-1w and N3-4w) were used in this study. All images were taken at the same magnification, and the total number of cells in each image were counted and categorized as those with nuclear staining with mEm48, and those with nuclear staining that contained one to five, six to 10 and more than 10 NIs.

### Western Blot Analysis

Total proteins were extracted from TrES1 cells and equal amounts (20-30 μg) of protein extract were loaded into a 9% polyacrylamide gel (Bio-Rad). Following electrophoresis, proteins were transferred onto a PVDF membrane followed by blocking in 5% skim milk for two hours. The membrane was then incubated with primary antibodies, mouse mEM48 (1:50), and γ-tubulin (1:2000; Sigma), followed by secondary antibody conjugated with peroxidase (Jackson Immuno Research Laboratories) for detecting proteins with a Western Lightning Chemiluminescence Reagent Plus. (PerkinElmer).

### In vivo differentiation of TrES1 and formation of teratoma in SCID mice

Undifferentiated TrES1 cell clumps were collected mechanically. TrES1 derived NPCs at N2 stage were collected by brief treatment with 0.05% trypsin/EDTA (Invitrogen) to produce single cell suspension. An estimate of 1 × 10^5 ^undifferentiated TrES cells and NPCs were resuspended in 5 ul of DPBS and implanted into the striatum of SCID mice. At four to 10 weeks after implantation, animals were euthanized, and the brain was recovered for further analysis. All surgical and animal procedures were approved by YNPRC/Emory Animal Care and Biosafety Committees. For staining of neuronal markers, the sections were incubated with primary antibodies (nestin, βIII tubulin, MAP2; Chemicon) at 4°C over night followed by thorough washes. A secondary antibody conjugated with Alexa Red (Molecular Probe) was used for detection of the primary antibodies. DNA was counterstained with Hoechst 33342 (5 μg/ml). The specimen was examined with an epifluorescent microscope.

### Immunhistochemical staining of mutant htt

Mice were anesthetized and perfused using 4% paraformaldehyde (PFA). Brain tissues were post-fixed in 4% PFA overnight at 4°C, transferred to 30% sucrose at 4°C, embedded in Optimal Cutting Temperature (OCT) medium (Sakura), cut at 50 μm, followed by DAB staining. Sections were incubated with 0.3% H_2_O_2 _for 15 mins, blocked for 1 hr, and incubated with mEM48 (1:50) at 4°C overnight. After washing with DPBS, the brain sections were processed with avidin-biotin using the Vectastain Elite ABC kit (Vector Laboratories), and immediately stained with DAB (Vector Laboratories) for 30-40 secs. Brain sections were mounted on the slides with mounting media (Sigma), and images were examined and captured by MetaMorph software (Universal Imaging).

### Statistical analysis

*Student t-test *was used for statistical analysis. Differences of *P *< 0.05 were considered statistically significant.

## Authors' contributions

CL: Embryo manipulation, establishment and characterization of TrES1, ECHC: Characterization of TrES1, PHC: Molecular analysis, BRS: Cell implantation and immunohistochemical analysis, SHY: ART in monkeys, ZJ: Genetic identity analyses, CL: Cell count and statistics, HCK: Technical support on stem cell techniques, RP: Experimental design, AWSC: Derivation of TrES1, experimental design and manuscript preparation. All authors read and approved the final version of the manuscript.
